# Acceptability, Precision and Accuracy of 3D Photonic Scanning for Measurement of Body Shape in a Multi-Ethnic Sample of Children Aged 5-11 Years: The SLIC Study

**DOI:** 10.1371/journal.pone.0124193

**Published:** 2015-04-28

**Authors:** Jonathan C. K. Wells, Janet Stocks, Rachel Bonner, Emma Raywood, Sarah Legg, Simon Lee, Philip Treleaven, Sooky Lum

**Affiliations:** 1 Childhood Nutrition Research Centre, UCL Institute of Child Health, London, United Kingdom; 2 Respiratory, Critical Care and Anaesthesia section (Portex Unit), UCL Institute of Child Health, London, United Kingdom; 3 UCL Department of Computer Science, Malet Place, London, United Kingdom; University of Bremen, GERMANY

## Abstract

**Background:**

Information on body size and shape is used to interpret many aspects of physiology, including nutritional status, cardio-metabolic risk and lung function. Such data have traditionally been obtained through manual anthropometry, which becomes time-consuming when many measurements are required. 3D photonic scanning (3D-PS) of body surface topography represents an alternative digital technique, previously applied successfully in large studies of adults. The acceptability, precision and accuracy of 3D-PS in young children have not been assessed.

**Methods:**

We attempted to obtain data on girth, width and depth of the chest and waist, and girth of the knee and calf, manually and by 3D-PS in a multi-ethnic sample of 1484 children aged 5–11 years. The rate of 3D-PS success, and reasons for failure, were documented. Precision and accuracy of 3D-PS were assessed relative to manual measurements using the methods of Bland and Altman.

**Results:**

Manual measurements were successful in all cases. Although 97.4% of children agreed to undergo 3D-PS, successful scans were only obtained in 70.7% of these. Unsuccessful scans were primarily due to body movement, or inability of the software to extract shape outputs. The odds of scan failure, and the underlying reason, differed by age, size and ethnicity. 3D-PS measurements tended to be greater than those obtained manually (p<0.05), however ranking consistency was high (r^2^>0.90 for most outcomes).

**Conclusions:**

3D-PS is acceptable in children aged ≥5 years, though with current hardware/software, and body movement artefacts, approximately one third of scans may be unsuccessful. The technique had poorer technical success than manual measurements, and had poorer precision when the measurements were viable. Compared to manual measurements, 3D-PS showed modest average biases but acceptable limits of agreement for large surveys, and little evidence that bias varied substantially with size. Most of the issues we identified could be addressed through further technological development.

## Introduction

Information on body size and shape is used to interpret many aspects of physiology in children, including nutritional status, cardio-metabolic risk and lung function [1–5]. Measurements of regional body girths or diameters can provide detailed information on the size of specific body components such as the limbs, abdomen and chest. Such data have traditionally been obtained through manual anthropometry, and are considered relatively reliable, and acceptable to the subject, in most age groups. However, manual anthropometry becomes time-consuming if multiple outcomes are desired, and the measurement of multiple dimensions (e.g. girths and diameters) requires different equipment.

A new alternative approach for the assessment of body size and shape comprises three dimensional photonic scanning (3D-PS) [6, 7]. This technique projects lasers or light stripes on to the body surface, and cameras record the distortion of these light patterns. Computer algorithms then reconstruct the 3D body surface topography, allowing automatic landmarks to be located through customised software [8, 9]. From these landmarks, a variety of girths, diameters and volumes can be derived using further algorithms [6, 7, 10]. 3D-PS offers a number of advantages over manual methods of shape assessment. First, raw data collection is extremely rapid, lasting only a few seconds. Second, a wide variety of digital shape outputs can be extracted through different software programs, and scans can be electronically archived for analysis with improved software in the future. Third, digital outputs can extend to 2D or 3D format, whereas manual measurements (e.g. girths) are only 1D. Fourth, composite models of shape can also be constructed, allowing the topographical location of shape change to be identified by accumulating scans over time [6, 7], though the need to adjust for growth makes this more challenging in children. In recognition of these benefits, several large surveys have utilised 3D-PS to acquire 1D and 2D body shape outputs in adults, and have shown substantial shape variability in association with age, gender and ethnicity [9–14].

Since young children are unlikely to tolerate large numbers of manual measurements, 3D-PS could be especially valuable in this age group. Within any population, children vary substantially in size and shape, indicating variability in body proportions and the distribution of organ, muscle and fat tissue [15, 16]. Such variability in regional shape may prove a better marker of traits such as abdominal obesity or lung function than simple size indices such as height or body mass index (BMI). Furthermore, body shape differences between ethnic groups are already established at young ages [17–19]. In adults, ethnic differences in body shape are an important factor contributing to variability in cardio-metabolic risk and physiological function [20–22]. Less is known about this scenario in children, but ethnic differences in body composition, even after standardising for BMI, have been reported [17, 18]. Indeed, the limits of BMI as a proxy for body composition in children, particularly in multi-ethnic populations, are well established [15, 17, 21]. However, as yet, 3D-PS has received minimal application in younger age groups, and its acceptability and quality of performance have not been evaluated. Two studies have reported validation data for 3D-PS versus manual measurements in adults, showing a high level of ranking consistency between the methods, but with systematic bias, where 3D-PS provides slightly larger values than manual measurements [9, 23]. Equivalent data for children are required.

We therefore sought to evaluate the acceptability, technical success, precision and accuracy of 3D-PS in a multi-ethnic sample of children aged 5–11 years. This work was part of the Size and Lung function In Children (SLIC) study, which was conducted in London, UK to investigate sources of variability in children’s lung function [24].

## Methods

The SLIC study is an epidemiological study conducted in London primary schools, aiming to explore ethnic differences in lung function in a young, multi-ethnic population of 5–11 year old children. The study was granted ethical approval by the London-Hampstead Research Ethics Committee (REC 10/H0720/53). All children with written informed parental consent were eligible to participate.

Children were grouped into four ethnic groups, “White” which included European, Hispanic or Latino, and Middle Eastern children; “Black” which included African and Caribbean children; “South Asian” which included Indian, Pakistani, Sri Lankan and Bangladeshi children; and “Other” which included children of any other ethnicities such as Chinese or Filipino, as well as children of mixed ethnic ancestry (e.g. Mixed South Asian / White).

Two indices on social deprivation were obtained. The Index of Multiple Deprivation 2010 (IMD2010) is a UK government-derived assessment assigned by post-code, based on a weighted amalgamation of income deprivation; employment deprivation; health deprivation and disability; education deprivation; crime deprivation; barriers to housing and services deprivation; and living environment deprivation. It is categorised in quintiles, with the first quintile being the least deprived. The Adapted Family Affluence Scale is a 0–6 point scale which is based on whether the child has their own bedroom, and how many vehicles and computers the child’s family has. Differences in age and social deprivation were tested by ANOVA, with Bonferroni correction.

### Measurement of body size and proportions

Attempts were made to measure body size and proportions using both manual measurements and 3D-PS in all children who were recruited during the first year of data collection for the SLIC study (October 2011- July 2012). Manual body size measurements included weight, height, girths of the chest, waist, knee and calf, and width and depth of the chest and waist. All these anthropometric measurements were performed using established protocols, as previously described [24]. Duplicate manual measures were obtained, and the average used in subsequent analyses.

Duplicate 3D body scans were attempted using NX16 instrumentation ([TC]^2^, Cary, North Carolina), the same technology used in previous surveys of adult shape [9, 10, 12]. The scanner was installed in a customised trailer, which was driven to a series of schools in order to collect data during school hours. Parents were not present during the scanning procedure.

**Fig 1:**
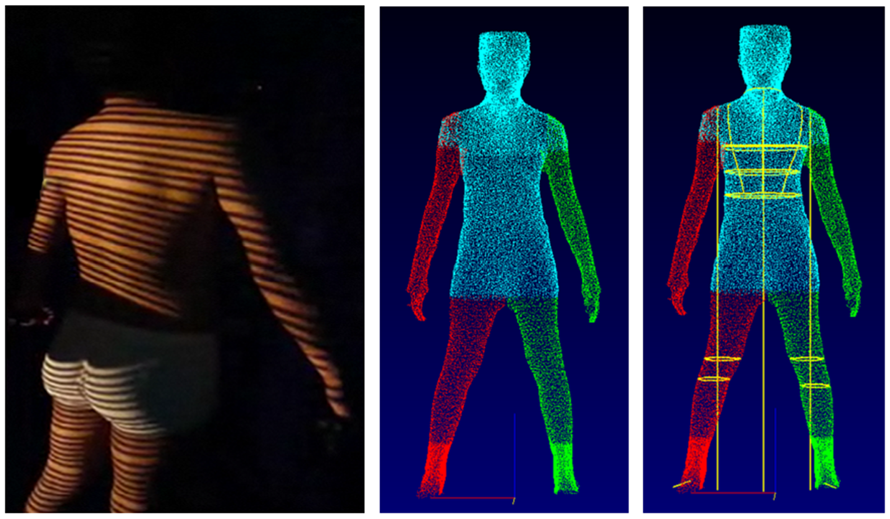
Stages in the generation of 3D-PS outputs. Left panel: projection of white light strips on the body surface, allowing reconstruction of skin surface topography through computer algorithms. Centre panel: raw body image extracted by computer algorithms. Right panel: electronic tape measure applied, following automatic extraction of key body landmarks.

The subjects wore form-fitting clothing and adopted a standardised position, with their feet located on landmarks on the scanner floor. The scanner projects strips of safe ‘white light’ (in the visible part of the electromagnetic spectrum) onto the body form and records the distortions induced by the body’s surface topography using 6 non-moving cameras over a period of ~8 seconds (Fig 1).

The scanner obtains a 3D ‘point cloud’ of raw photonic data and then reconstructs the skin topography using computer algorithms. More detailed descriptions of this process have been published elsewhere [8, 25]. The software used was Body Measurement System Version 5.3; [TC]^2^). For our study, the software automatically extracted girths, widths and depths of the chest and waist, and girths of the knee and calf on both sides of the body. The left and right knee and calf data were averaged for use in subsequent statistical analyses. Either one or two scans were used to extract 3D outcomes, according to whether they were considered technically successful.

### Acceptability and feasibility

Acceptability was assessed in terms of the numbers invited to undergo 3D scans, and their participation rates, while feasibility was assessed in terms of successful scan extraction rates. We calculated the frequency of those with parental consent, who refused to undergo the scan, and the frequency of technical failure in those who agreed to be scanned.

We further categorised technical failure as follows: movement during the scan; interference by clothing (clothing artefact); failure of the software to extract a raw body image; and failure of the software to apply automatic landmarks and extract digital shape outputs. In order to stimulate further work to improve the technical performance of 3D-PS in children, examples of specific problems were selected for illustration. Between-group variability in scanning success rates, and in the prevalence of specific causes of technical failure, was calculated using odds ratios obtained using logistic regression. In these models, we also took into account age, sex and the indices of social deprivation.

### Precision

Precision was calculated from duplicate scans when available. In some instances, a third scan was obtained at the time of measurement due to concern over the quality of one of the two original scans. The mean of two or three scans was used when comparing methods. However, in calculating precision we discarded data from children with 3 scans, as this approach inflates precision. Using only duplicate scans, we calculated precision using the following equation, given by Bland and Altman [26]:

$$Precision\;=\;\;\sqrt{sum\;of\;squared\;differences\;between\;scans/n}$$

### Agreement between methods

Agreement was assessed using the method of Bland and Altman [26], which calculates the mean bias between techniques along with its limits of agreement, which are obtained as twice the standard deviation of the bias. This bias was tested for significant difference from zero by paired t-test. We also used correlation analysis to establish whether the bias varied in relation to the mean value obtained using both methods, i.e. whether bias was associated with the magnitude of the outcome [26]. We further regressed 3D outputs on manual outputs, in order to derive the intercept and slope. Where the intercept differed from zero, outputs from one technique differed systematically in magnitude from the other. Where the slope differed significantly from 1, the two techniques showed inconsistent agreement across the range of size.

All analyses were conducted in Excel or SPSS. A cut-off of p = 0.05 was used for statistical significance.

## Results

**Fig 2:**
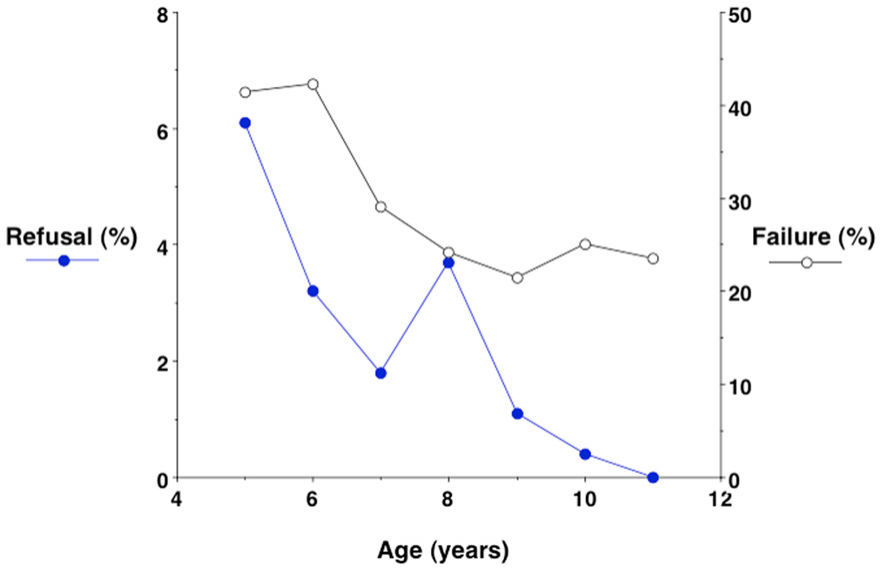
Age-associated reductions in the percentages of children refusing to be scanned, or producing scans that were not technically successful. The rate of refusal fell steadily from 6% at 5 years to zero at 11 years, whereas the percentage of failed scans decreased with age, but remained ~25% even in the older age groups.

Parental consent for their child to undergo 3D-PS was obtained for 94.8% (1496/1578) of those approached. Amongst these 1496 children, a scan was not attempted in 12 (0.8%), as the equipment was not ready, or the child was not available at the appropriate time. These 12 children were not included when calculating acceptability or feasibility. Of the remaining 1484 children, 38 (2.6%) refused, giving an acceptability rate of 97.4% (Table 1). Those refusing were younger (Δ = 1.0 (SE 0.3) y; p<0.0001), shorter (Δ = 6.8 (SE 1.9) cm; p<0.0001) and had lower BMI (Δ = 1.4 (SE 0.5) kg/m^2^; p<0.0001) than those agreeing to be scanned (Table 2), but the differences in body size were no longer significant after adjustment for age. There was no ethnic difference in the likelihood of refusal. The age-associated reduction in the percentage of children refusing is illustrated in Fig 2.

**Table 1:** Feasibility in performing 3D photonic scans in young children in a field setting.

Outcome	Whole sample		Ethnicity							
	N		White		Black		South Asian		Other	
			N		N		N		N	
Consented	1496		563		442		297		194	
	N	%	N	%	N	%	N	%	N	%
Not tested *	12	0.8	3	0.5	2	0.5	4	1.3	3	1.5
Refused ‡	38	2.6	14	2.5	10	2.3	11	3.8	3	1.6
Technical failure # $	424	29.3	141	25.8	162	37.7	82	29.1	39	20.7
Measured successfully # $	1022	70.7	405	74.2	268	62.3	200	70.9	149	79.3

* Rate calculated as proportion of those consented

^‡^ Rate calculated as proportion of those invited to be scanned

^#^ Rates calculated as proportion of those actually scanned

^$^ Significant difference between ethnic groups by Chi-square test p<0.0001

**Table 2:** Age and size by scan outcome in those who underwent a scan.

Outcome	n	Age (years)		Height (cm)		BMI (kg/m^2^)	
		Mean	SD	Mean	SD	Mean	SD
Successful	1022	8.44	1.57	131.8	11.2	17.5	3.2
Refused	38	7.05 ‡	1.57	124.1 ‡	9.0	16.0 ‡	2.6
Technical failure	424	7.85 ‡	1.67	130.6 *	11.7	17.4	3.4

* Significant difference from successes (p<0.05)

^‡^ Significant difference from successes (p<0.001)

There was no difference in age between the ethnic groups by ANOVA. Scores for the Index of Multiple Deprivation were significantly greater (p<0.0001) in the three ‘non-White’ groups relative to the ‘White’ children (3.27 SD 1.36). The ‘Black’ children (4.46, SD 0.85) further had scores significantly greater (p<0.0001) than the ‘South Asian’ (3.83, SD 0.99) and ‘Other’ (3.78, SD 1.32) groups. For the Family Affluence Scale, the ‘White’ (3.70, SD 1.42) and ‘Other’ (3.72, SD 1.40) children had significantly higher (p<0.0001) scores than the ‘South Asian’ children, 3.15, SD 1.33), and all three of these groups had significantly higher (p<0.005) scores than the ‘Black’ children (2.88, SD 1.25).

On average, the duration for performing detailed manual anthropometry was ~10 minutes per child. Although each 3D-PS measurement can be captured in 8 seconds, the preparation and processing time for this assessment, which includes undressing and dressing, encouraging the child to stand still and having to repeat the scan (if child willing) to achieve at least 2 acceptable scans also took an average of 10 minutes per child.

Of the 1446 children who underwent a scan, the process was unsuccessful in 29.3%, due to problems with clothing, body movement, or failure to extract a viable body model. Thus, the technique proved technically feasible in 70.7%. Adjusting for age, the odds of technical failure were higher in ‘Black’ children (1.84; 95%CI 1.34, 2.51; p<0.0001) relative to ‘White’ children, however the other two groups showed no difference. The two measures of social deprivation were not significant in this model.

Aside from ethnic variability, the risk of scan failure was associated with the child’s age and body size (Table 2), but not with sex. The mean age of those scanned successfully was 0.7 years (p<0.0001) greater than those whose scans were not successful. Adjusting for age, the mean weight and height of those not scanned successfully were 1.7 kg and 3.3 cm lower respectively (p<0.0001) than those scanned successfully, however there was no significant difference in BMI. The age-associated reduction in the percentage of children with unsuccessful scans is illustrated in Fig 2.

Categorisation of the reasons for scan failure is given in Table 3. Among reasons for technical failure, the prevalence of excessive body movement was relatively low (2.8% of all those scanned, and 9.4% of all failed scans). Approximately equal proportions (8 to 9%) of scans overall failed because of clothing artefact, failure of the software to extract a body image, and failure of the software to locate landmarks for shape outputs. The challenge of extracting a body image was significantly more common amongst the ‘Black’ and ‘South Asian’ children, while the likelihood of clothing artefact was greater in the ‘White’ children. A small proportion of scans failed when the trailer was sensitive to vibrations from nearby busy roads.

**Table 3:** Reasons for scan failure in the whole sample and by ethnicity.

Reason for failure	Total			White			Black			South Asian			Other		
	No	%1	%2	No	%1	%2	No	%1	%2	No	%1	%2	No	%1	%2
Clothing artefact	126	8.7	29.7	61	11.2	43.3	29	6.7	17.9	24	8.5	29.3	12	6.4	30.8
Failed to create body image	127	8.8	29.9	31	5.7	22.0	60	14.0	37.0	28	9.9	34.1	8	4.3	20.5
Failed to extract 3D outcomes	115	8.0	27.1	36	6.6	25.5	41	9.5	25.3	22	7.8	26.8	16	8.5	41.0
Movement during scanning	40	2.8	9.4	5	0.9	3.5	26	6.0	16.0	7	2.5	8.5	2	1.1	5.1
Other (system failure, fear of dark)	16	1.1	3.8	8	1.5	5.7	6	1.4	3.7	1	0.4	1.2	1	0.5	2.6
Total	424	29.3	100	141	25.8	100	162	37.7	100	82	29.1	100	39	20.7	100

%1—percentage of all children scanned in this group

%2—percentage of failed scans in this group

**Fig 3:**
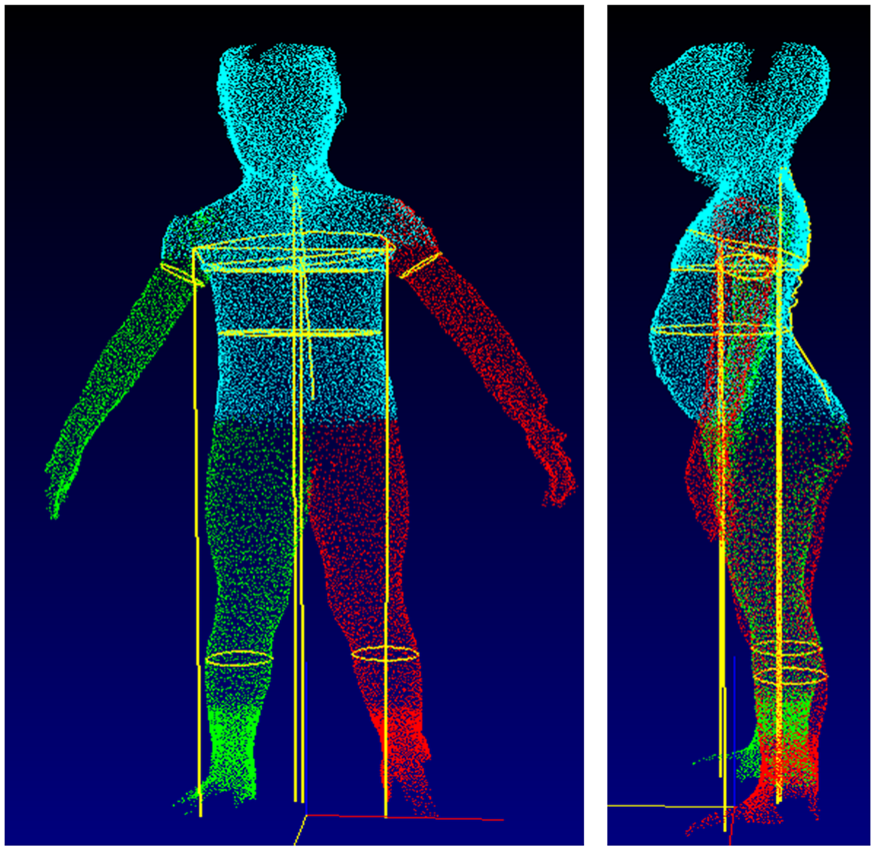
Scan characterised by technical failure, due to inability to extract chest girth data because of atypical posture. However, the raw scan was of good quality, indicating that the problem lies in the software.

**Fig 4:**
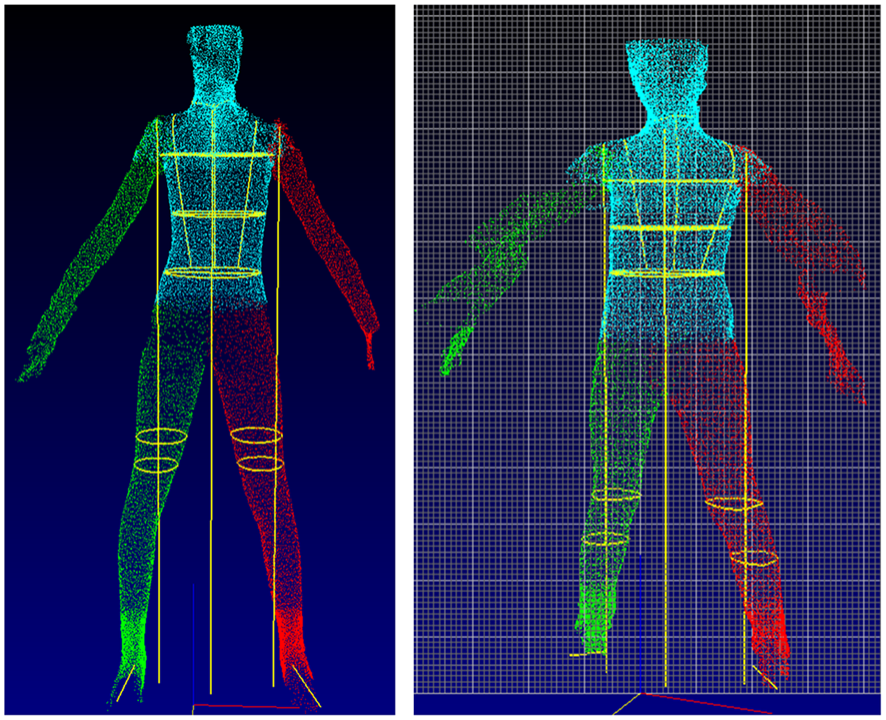
Scans characterised by technical failure, due to inability to locate leg girths (left hand panel) and movement of the arms during the scanning process (right hand panel).

**Fig 5:**
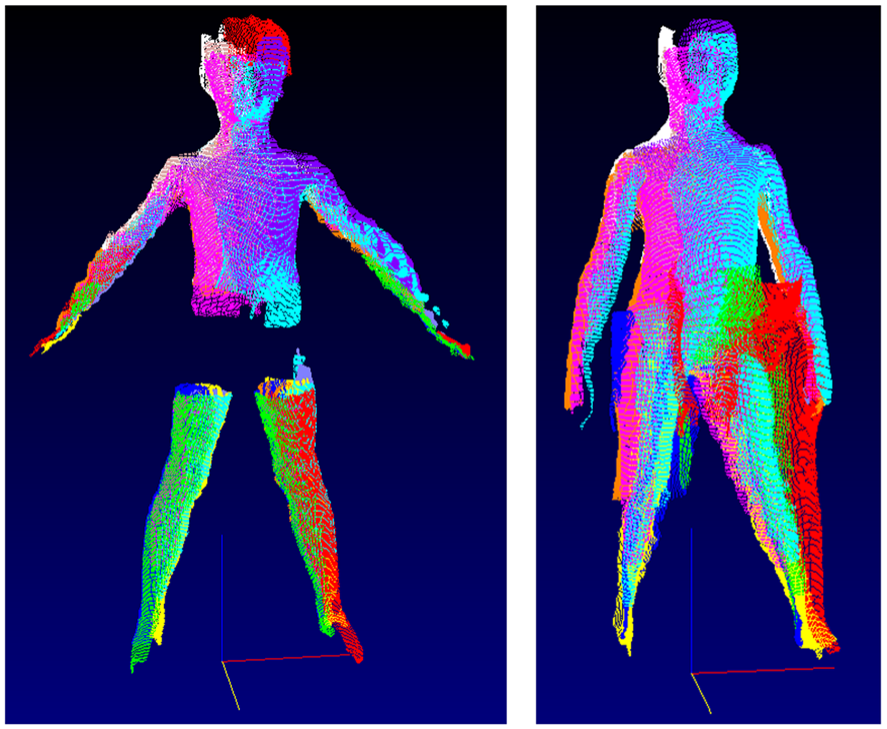
Scans characterised by technical failure, due to artefacts from inappropriate clothing (left hand panel) and movement of the scanning truck (right hand panel).

Examples of technical failures are illustrated, including inability to extract chest data due to atypical posture (Fig 3), failure to locate leg girths and body movement of the arms (Fig 4), and artefacts arising from inappropriate clothing and vibrations affecting the scanning truck (Fig 5).

Precision of duplicate measurements by 3D-PS and by manual measurement is given in Table 4. Duplicate successful 3D scans were available for 589 children, 57.6% of those in whom 3D-PS was technically successful overall. The paired measurements by 3D-PS are illustrated for each outcome in supporting information file S1 Fig. The magnitude of precision in the manual measurements was better by at least a factor of 10 than that from 3D-PS. For example, 3D-PS precision of waist and chest girth was ~1.5 cm, whereas manual precision was ~0.1 cm. Height was not compared between methods as it is not extracted by 3D-PS.

**Table 4:** Mean, variability and precision of shape outputs by 3D-PS and manual methods.

	3D measures			Manual measures			P-value for difference between means
Outcome	Mean	SD	Precision	Mean	SD	Precision	
Chest girth (cm)	68.7	8.8	1.57	65.1	8.0	0.13	<0.0001
Chest width (cm)	23.9	2.8	0.84	21.9	2.6	0.06	<0.0001
Chest depth (cm)	17.4	2.3	0.69	15.6	1.9	0.07	<0.0001
Waist girth (cm)	59.7	8.3	1.49	58.4	7.9	0.06	<0.0001
Waist width (cm)	20.7	2.7	0.60	19.5	2.6	0.06	<0.0001
Waist depth (cm)	16.2	2.4	0.67	15.5	2.3	0.06	<0.0001
Knee girth (cm)	29.7	3.8	1.36	28.2	3.4	0.06	<0.0001
Calf girth (cm)	27.4	3.5	0.90	26.9	3.4	0.04	<0.0001

**Fig 6:**
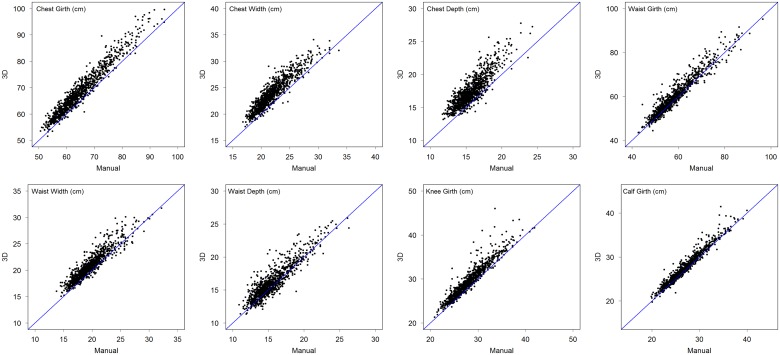
Plots of 3D-PS values (y-axis) against manual values (x-axis) for each of the eight outcomes, superimposed on the line of identity. The plots illustrate a general tendency for 3D-PS values to exceed manual values, with this over-estimation varying between outcomes. The magnitude of 3D-PS bias is described in detail in Table 5 and supporting information file S1 Table.

The scatter of 3D-PS values against manual values is shown for each outcome in Fig 6, while Table 5 shows correlations between 3D-PS and manual measurements in the whole sample, and by ethnicity. The majority of correlations were >0.90, and all were ≥0.84. This indicates relatively high consistency in ranking, with variability in 3D-PS outcomes explaining ~75% to ~85% of variability in torso depths and widths, and ~92% to ~97% of the variability in body girths.

**Table 5:** Correlations between 3D-PS and manual outcomes for the whole sample and for each ethnic group.

Outcome	All	White	Black	South Asian	Other
	r	r	r	r	r
Chest girth (cm)	0.97	0.96	0.97	0.98	0.97
Chest width (cm)	0.92	0.91	0.93	0.94	0.91
Chest depth (cm)	0.87	0.84	0.86	0.90	0.87
Waist girth (cm)	0.96	0.95	0.96	0.97	0.96
Waist width (cm)	0.93	0.91	0.94	0.93	0.93
Waist depth (cm)	0.90	0.89	0.88	0.93	0.92
Knee girth (cm)	0.95	0.92	0.96	0.93	0.92
Calf girth (cm)	0.97	0.97	0.97	0.98	0.98

All correlations p<0.0001

Table 6 shows Bland-Altman statistics for the assessment of agreement between techniques. There was a significant bias between 3D-PS and manual measurements for all outcomes, in all cases the 3D-PS outcomes being larger. These biases were furthermore significantly associated with the mean of the measurement by the two methods, indicating that bias varied with outcome size. There were negligible ethnic differences in these biases and their correlations with size: chest girth bias was significantly smaller in the ‘White’ than the ‘South Asian’ or ‘Other’ group; and the ‘White’ group had smaller bias in chest width, waist depth and knee and calf than the ‘Black’ group. In all cases, these ethnic differences were of small magnitude.

**Table 6:** Biases between 3D-PS and manual outcomes.

	Bias (3D-Manual)			Correlation	
Outcome	Mean	SD	p-value	R	p-value
Chest girth (cm)	3.67	2.23	<0.0001	-0.13	<0.0001
Chest width (cm)	1.96	1.09	<0.0001	0.21	<0.0001
Chest depth (cm)	1.75	1.15	<0.0001	0.39	<0.0001
Waist girth (cm)	1.36	2.37	<0.0001	0.14	<0.0001
Waist width (cm)	1.15	1.01	<0.0001	0.08	0.013
Waist depth (cm)	0.60	10.5	<0.0001	0.11	<0.0001
Knee girth (cm)	1.39	1.21	<0.0001	0.29	<0.0001
Calf girth (cm)	0.62	0.80	<0.0001	0.15	<0.0001

Supporting information file S1 Table provides additional information on the level of agreement between techniques for the whole sample, by providing intercepts and slopes for the regression of 3D-PS measurements on manual measurements. These clarify that 3D-PS outcomes were systematically greater (intercept significantly >1) for all outcomes except girths of the chest, knee and calf. However, slopes differed significantly from 1 only for chest girth and depth and knee girth, indicating that the bias varied over the magnitude of the outcome.

## Discussion

The speed of data collection per subject of 3D-PS potentially makes it a valuable resource for collecting multiple anthropometric outcomes in very large surveys, but these advantages are offset by the need for expensive equipment, and currently by lack of evidence regarding its viability and accuracy. In adults, 3D-PS measurements has shown high ranking consistency with manual anthropometry, although 3D-PS provides slightly but systematically larger values [9, 23]. Acceptability of the technique has not been formally assessed, but large samples took part in national sizing surveys in several countries [9, 10, 12]. Our study represents the first effort to assess the acceptability, feasibility, precision and accuracy of 3D-PS in children, using a large multi-ethnic sample aged 5–11 years. This information will help develop this method for future application in pediatric research and clinical practice.

### Acceptability

The acceptability proved high across the entire sample, with only 2.6% of those invited to participate declining. The rate of acceptance might have been even higher had parents of younger children been present. Those who declined, or refused, tended to be younger than those who accepted and were scanned successfully. Nevertheless, the vast majority of all age groups underwent a scan, and the main challenge that we encountered in this study was not with children’s acceptance but with technical aspects of 3D-PS, with 29.3% failing to produce a viable scan.

### Technical problems

Several generic reasons for technical failure were documented, some pertaining to the practical challenges of scanning young children, and others relating to technical limitations of the current hardware and software.

Even after taking age into account, those in whom the scan was not successful tended to be slightly shorter and lighter, indicating that the software is marginally less successful in extracting data from smaller children. Nevertheless, the majority of scans at all ages were successful, as indicated in Fig 2.

Problems with clothing affected 8.7% of the whole sample, and were more common amongst the ‘White’ children. This problem could be resolved by offering standardised form-fitting clothing, although a minority of children may still be unwilling to comply due to sensitivity concerning body shape, or cultural variability regarding modesty. Some scans failed due to extreme clothing colour, an issue that again could be resolved by making available appropriate clothing, as was done in the adult surveys.

Body movements must be anticipated in young children. Adults undergoing 3D-PS have the advantage of a stabilising handhold to aid maintenance of an ideal posture during assessments. However in this study, attempts to adapt the handhold for children were unsuccessful and will need manufacturer’s input to customise the handhold for children to minimise body movements. The effect of such movements on scan quality could potentially also be reduced, by shortening the duration of the scan. This could be achieved in part through physical alterations to the scanner: since children are shorter than adults, the cameras do not need to descend from as great a height above the floor. Other options are also available, such as speeding up the capture of photonic data. Thus, technical development could potentially resolve this problem.

Failure of the software to extract body shape or body measurements can be attributed to insufficient tailoring of the computer algorithms to the range of variability in children’s shape. The software we used was originally developed for application with adults, and therefore lacks ‘training’ on younger age groups. The likelihood of failure to extract outcomes varied by ethnicity, suggesting that such training must be done on multi-ethnic datasets. There was no indication that ethnic differences in technical failure were associated with age or indices of social deprivation, suggesting that ethnic variability in posture or physique was the primary challenge. However, there were also minor differences between ethnic groups in the likelihood of body movement. Overall, this source of error could likewise be minimised through further development of 3D-PS software algorithms.

When the trailer housing the scanner was parked near busy roads, vibrations could interfere with data capture. This problem could potentially be resolved by increasing the stability of the mobile unit. From the opposite perspective, the mobile unit proved very valuable for introducing the technology in standardised format into a number of schools.

Overall, the relatively high failure rate of 3D-PS in young children compared to adults can be attributed to issues that should be possible to resolve through technological development. For example, if we had resolved all technical problems with scan extraction, and encountered only problems of body movement and clothing artifact, the success rate would have been 87%. The feasibility problems we encountered relate primarily to a specific version of the technology we used, rather than universal limitations of 3D-PS.

The time required to complete 3D-PS was similar to that required to collect equivalent manual data. If greater numbers of shape outcomes were required, 3D- PS would quickly become more time-effective, whilst technical changes could also speed up data collection. These issues are often very important in large surveys, and may have staff cost implications. Height and sitting height are not extracted by 3D-PS, and must be obtained manually, requiring additional time and equipment. However, height is routinely measured in clinical practice, hence manual measurement of this outcome is relatively undemanding. On the other hand, the initial cost of equipment is substantially greater for 3D-PS than manual approaches, hence any cost-benefits of 3D-PS are likely to restricted to large surveys.

### Precision and accuracy

Precision of 3D-PS was poorer by a factor of 10 than that of manual measurements, which is likely to arise for several different reasons. Manual measurements are conducted with much greater control over the subject, as the investigator can ask the child to adopt and hold a given posture, potentially increasing the likelihood of making identifying the landmark correctly. However, this may also artificially inflate precision of manual anthropometry as it is human nature to return to the same landmarks when collecting duplicate data. 3D-PS must identify body landmarks automatically *de novo* each scan, and subtle changes in posture between repeated scans may confound this process. Despite this difference between techniques, 3D-PS achieves a level of precision that is adequate for large surveys, in which the aim is to rank individuals in terms of population variability in size and shape outcomes.

Using manual measurements as the gold standard reference method, the accuracy of 3D-PS showed a relatively consistent bias to larger measurements, although the magnitude of this difference was always modest. This is consistent with previous validation studies, and can be attributed to the fact that manual measurements involve a slight tension, as the tape is held over the skin [23]. For example, disagreement between manual and 3D measurements decreased when a mannequin was measured instead of a human body [23]. The limits of agreement were of intermediate magnitude, as visually evident in Fig 6. While the correlations indicate relatively good ranking consistency between techniques, the level of agreement within individual children was wider than would be acceptable for studies where, for example, the aim was to identify children above or below specific cut-offs.

These biases and limits of agreement are of sufficient magnitude for us to conclude that the techniques cannot be regarded as interchangeable. As yet, it is unclear what proportion of the bias can be attributed to different body locations being measured, as opposed to inaccuracy of the software algorithms, and further work is needed to address this. In any comparison using the Bland-Altman method, it must be remembered that both techniques contribute to the lack of agreement, however in this case the main source of imprecision was clearly 3D-PS.

Overall, we suggest that 3D-PS shows great promise as a new digital tool for anthropometric surveys, but we have outlined a number of issues that will require attention in order to improve the quality of the data.

## Conclusions

In summary, our study of 3D-PS showed that the technique has great promise for further work in the assessment of children’s size and shape in epidemiological research. The technique had poorer technical success than manual measurements, and had poorer precision when the measurements were viable. Using manual measurements as the reference, 3D-PS showed modest average biases but acceptable limits of agreement for large surveys, and little evidence that the bias varied substantially with size. There were subtle differences between ethnic groups in the rate of success of 3D-PS, but very little indication that accuracy and precision varied by ethnicity. We applied a version of 3D-PS developed for application with adults, and most of the issues we identified could be addressed through further technological development.

## Supporting Information

S1 Fig Paired measurements by 3D-PS. (TIFF)

S1 Table Regressions of 3D outcomes on manual outcomes to test whether intercepts differ from zero, and slopes differ from 1. (DOCX)
