# Supplementary figures and images for: Acceptability, Precision and Accuracy of 3D Photonic Scanning for Measurement of Body Shape in a Multi-Ethnic Sample of Children Aged 5-11 Years: The SLIC Study
Source: PLoS One. 2015 Apr 28;10(4):e0124193. doi: 10.1371/journal.pone.0124193 (PMC4412635; doi:10.1371/journal.pone.0124193)

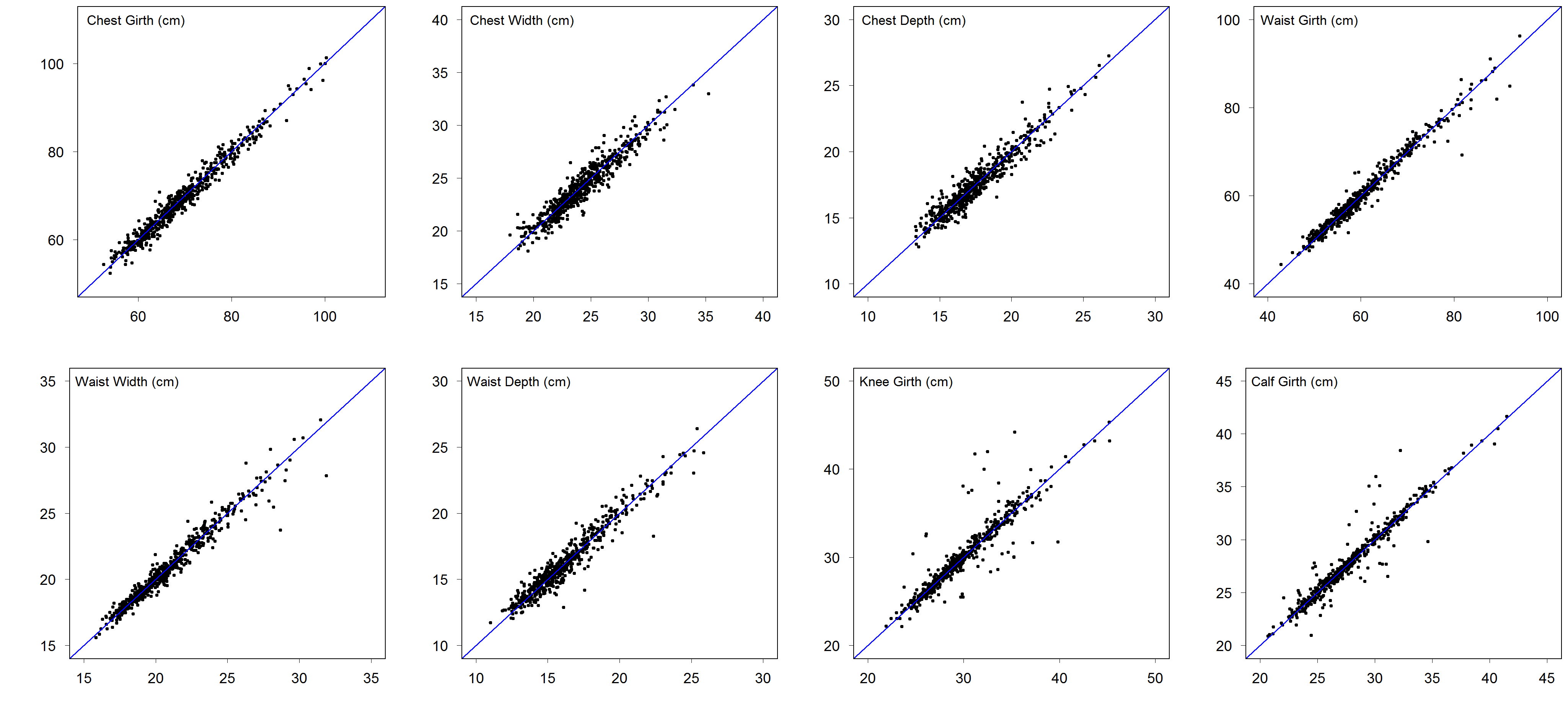

Supplement: S1 Fig — (TIFF) [file pone.0124193.s001.tiff]
