# Supplementary material for: Acceptability, Precision and Accuracy of 3D Photonic Scanning for Measurement of Body Shape in a Multi-Ethnic Sample of Children Aged 5-11 Years: The SLIC Study
Source: PLoS One. 2015 Apr 28;10(4):e0124193. doi: 10.1371/journal.pone.0124193 (PMC4412635; doi:10.1371/journal.pone.0124193)
Supplement: S1 Table — (DOCX) [file pone.0124193.s002.docx]

**Table S1. Regressions of 3D outcomes on manual outcomes to test whether intercepts differ from zero, and slopes differ from 1**

| 3D outcome | Intercept | | Slope | | Model fit | |
| --- | --- | --- | --- | --- | --- | --- |
|  | Coefficient | 95% CI | Coefficient | 95% CI | SEE | R^2^ |
| Chest girth (cm) | -0.88 | -1.97, 0.21 | **1.07** | **1.05, 1.08** | 2.16 | 0.94 |
| Chest width (cm) | **1.88** | **1.31, 2.44** | 1.00 | 0.98, 1.03 | 1.09 | 0.85 |
| Chest depth (cm) | **0.71** | **0.12, 1.31** | **1.07** | **1.03, 1.10** | 1.14 | 0.75 |
| Waist girth (cm) | **1.43** | **0.34, 2.51** | 1.00 | 0.98, 1.02 | 2.37 | 0.92 |
| Waist width (cm) | **2.03** | **1.56, 2.50** | 0.95 | 0.93, 0.98 | 1.00 | 0.86 |
| Waist depth (cm) | **1.40** | **0.67, 1.83** | 0.95 | 0.92, 0.98 | 1.04 | 0.82 |
| Knee girth (cm) | 0.17 | -0.48, 0.83 | **1.04** | **1.02, 1.07** | 1.20 | 0.89 |
| Calf girth (cm) | 0.37 | -0.04, 0.78 | 1.01 | 0.99, 1.02 | 0.80 | 0.95 |

Each 3D outcome predicted from the relevant manual outcome. Constants differing significantly from zero, and slopes differing significantly from 1, given in bold.

Slopes >1 for chest girth and depth and knee girth indicated a tendency for 3D-PS to exceed manual values at larger trait size, whereas slopes <1 for waist width and depth indicated the reverse trend, increasing disagreement at smaller size.
